# A20 (TNFAIP3) alleviates viral myocarditis through ADAR1/miR-1a-3p-dependent regulation

**DOI:** 10.1186/s12872-021-02438-z

**Published:** 2022-01-16

**Authors:** Bin Li, Xing Xie

**Affiliations:** Department of Cardiovascular Medicine, Affiliated Hospital of Xiangnan University, No. 25, West Renmin Road, Chenzhou, 423000 Hunan People’s Republic of China

**Keywords:** A20, miR-1a-3p, ADAR1, Dicer, Viral myocarditis, Inflammation, Apoptosis

## Abstract

**Objective:**

To investigate the effect of A20 and how A20 is regulated in viral myocarditis (VMC).

**Methods:**

BABL/C mice, primary neonatal rat cardiomyocytes and H9c2 cells were infected with Coxsackie virus B3 (CVB3) to establish animal and cellular models of VMC. H&E staining revealed the pathologic condition of myocardium. ELISA measured the serum levels of creatine kinase, creatine kinase isoenzyme and cardiac troponin I. The effects of A20, miR-1a-3p and ADAR1 were investigated using gain and loss of function approaches. ELISA measured the levels of IL-6, IL-18 and TNF-α in serum or cell culture supernatant. TUNEL staining and flow cytometry assessed the apoptosis of myocardium and cardiomyocytes, respectively. RNA-binding protein immunoprecipitation and dual-luciferase reporter assays verified the binding between A20 and miR-1a-3p. Co-immunoprecipitation assay verified the binding between ADAR1 and Dicer.

**Results:**

A20 was underexpressed and miR-1a-3p was overexpressed in the myocardium of VMC mice as well as in CVB3-infected cardiomyocytes. Overexpression of A20 suppressed cardiomyocyte inflammation and apoptosis in vivo and in vitro. miR-1a-3p promoted CVB3-induced inflammation and apoptosis in cardiomyocytes by binding to A20. The expression of miR-1a-3p was regulated by ADAR1. ADAR1 promoted the slicing of miR-1a-3p precursor by binding to Dicer.

**Conclusion:**

A20, regulated by ADAR1/miR-1a-3p, suppresses inflammation and cardiomyocyte apoptosis in VMC.

**Supplementary Information:**

The online version contains supplementary material available at 10.1186/s12872-021-02438-z.

## Introduction

Myocarditis is an inflammatory disease of the myocardium. Patients diagnosed with myocarditis most often present with nonspecific symptoms or acute cardiac involvement including dyspnea, chest pain, heart failure, palpitation and cardiac arrhythmia [1]. Myocarditis is associated with substantial morbidity and mortality, and has been identified as one of the causes of dilated cardiomyopathy [2]. Among various viral causes, enterovirus and parvovirus B19 are the most prevalent pathogens responsible for the outbreak of acute myocarditis [3]. Nevertheless, Coxsackie virus B3 (CVB3) remains the most extensively studied virus in clinical and experimental myocarditis. Persistence of viral infections predicts poor improvement and higher mortality of myocarditis patients, and clearance of virus can significantly improve left ventricular function [4]. Generally, viral infection induces lysis of myocardial cells, followed by maladaptive immune-mediated responses that kill virus-infected and uninfected cardiomyocytes [5]. However, the exact pathogenic mechanism of viral myocarditis (VMC) is far from being fully understood.

A20, also known as TNFAIP3, is well characterized as a nuclear factor (NF)-κB inhibitory and antiapoptotic protein, and regulates the innate immune response to pathogens [6]. Human genetic studies have associated single-nucleotide polymorphisms (SNPs) of the gene encoding A20 with the susceptibility to an extensive range of human diseases, including systemic lupus erythematosus, rheumatoid arthritis, psoriasis, type 1 diabetes, Crohn's disease, and systemic sclerosis [7]. These disease-associated SNPs might reduce the anti-inflammatory function of A20 by reducing its expression. A study by Gui et al. has demonstrated that A20 can mitigate CVB3-induced myocarditis by inhibiting the NF-κB signaling [8]. They later found that Astragaloside IV exerted therapeutic effects on CVB3-induced myocarditis by increasing the expression of A20 [9]. Up-regulation of Argonaute proteins (AGO1 and AGO3) inhibited the expression of TNFAIP3 in CVB3-induced myocarditis [10]. A20 shows the potential as a therapeutic target in VMC treatment, but how A20 is regulated in VMC is still elusive.

Studies have shown that A20 is a RNA-binding protein and can be targeted by different microRNAs (miRNAs). MiRNAs control over 50% of mammalian protein-coding genes, and show dysregulated expression in human diseases [11]. MiRNA-1a-3p (miR-1a-3p) is a less-discussed miRNA, and little is known about its function in VMC. Metformin protected cardiomyocytes against ischemia–reperfusion injury by decreasing the expression of miR-1a-3p [12]. Among selected cardiac- or muscle-specific miRNAs, miR-1a-3p was significantly down-regulated during postnatal heart growth [13]. Given the aforementioned information, miR-1a-3p is likely to play a negative role during the normal functioning of cardiomyocytes. ADAR1 is an important regulator of adenosine to inosine editing of RNA transcripts, and is implicated in the replication of viruses. Down-regulation of ADAR1 ameliorated CVB3-induced VMC in the early stage of viral infection while aggravated the disease progression in the middle-late stage by mediating inflammatory responses through protein kinase R (PKR) and NF-κB signaling [14]. ADAR1p150 formed a complex with Dicer to promote the expression of miR-222 in VMC [15]. Whether the ADAR1/Dicer complex can promote the activity of miR-1a-3p is not known. In this study, we validated the function of A20 and how it was regulated by ADAR1/Dicer/miR-1a-3p in VMC.

## Materials and methods

### Experimental animals and viruses

Male BABL/C mice (4–6 weeks, 16–19 g) were purchased from Shanghai SLAC Laboratory Animal Co., Ltd. (Shanghai, China). All animal experiments abided by the Regulations on the Management of Laboratory Animals and related ethic requirements. The mice were fed at 25 ± 2 °C with 60–80% humidity for one week, and provided with 12 h: 12 h light/dark cycles and standard food and water.

CVB3 used in this study was previously preserved in our laboratory. Hela cells (ATCC, Manassas, Virginia, USA) were used as vectors for replication of CVB3. Virus titer of CVB3 was measured by the TCD50 method.

### Mouse model of VMC

BABL/C mice were randomly divided into six groups (each group consisted of ten mice). The Normal group was injected with 100 μl of PBS. The other five groups were intraperitoneally injected with 100 μl of PBS-diluted CVB3 (10^3^ TCD50). Weight and death of the mice in the Normal group and in a CVB3 group were recorded every day. Seven days after injection, the mice were intraperitoneally injected with pentobarbital sodium (60 mg/kg) and ketamine (50 mg/kg), and killed by cervical dislocation. Cardiac tissue and blood were taken from the mice and preserved for later use.

### Injection of lentiviruses

Four groups of CVB3-injected mice were further injected with A20 knockdown lentiviruses (LV-sh-A20), A20 overexpression lentiviruses (LV-A20) or their negative controls (LV-sh-NC or LV-NC). The lentivirus vectors were synthesized by GenePharma (Shanghai, China). The mice were injected with 100 μl of lentiviruses (1 × 10^8^ TU/ml) in tail vein on day 2 and day 5 after infection of CVB3.

### Isolation of primary neonatal rat cardiomyocytes

Ventricles were taken from suckling rats within 24 h of birth through the chest that was opened along both sides. The ventricles were washed with PBS and cut into 1 mm^3^ patches. The ventricle patches were digested by 0.1% trypsin in turn to obtain cell suspension. The suspension was purified by differential centrifugation, and added into DMEM containing 10% fetal bovine serum to adjust the cell density (2 × 10^5^ cells/ml). Fibroblast proliferation was suppressed by 0.1 mol/l 5-bromodeoxyuridine. The cells were cultured at 37 °C with 5% CO_2_.

### Cellular model

Primary neonatal rat cardiomyocytes or H9c2 cells (ATCC, Manassas, Virginia, USA) were incubated with CVB3 (100 TCD50) in serum-free medium for 2 h. The medium was sucked out after the incubation. The cells were then cultivated in normal medium.

### Cell transfection

After CVB3 infection, primary neonatal rat cardiomyocytes and H9c2 cells were transfected with A20 knockdown or overexpression lentiviruses (LV-sh-A20 and LV-A20), miR-1a-3p inhibitor lentiviruses or their respective negative controls (LV-sh-NC, LV-NC or inhibitor NC). Uninfected primary neonatal rat cardiomyocytes and H9c2 cells were transfected with ADAR1 knockdown or overexpression lentiviruses (LV-sh-ADAR1 and LV-ADAR1), or the negative control (LV-sh-NC or LV-NC). All the lentiviruses were synthesized by GenePharma (Shanghai, China). The lentiviruses were added into l ml of cell suspension containing 2 × 10^5^ cells. The volume of lentiviruses varied from 10 to 200 μl depending on the cell type and lentivirus titration. After incubation with lentiviruses for 24 h, the cells were cultured in fresh normal medium.

### Statistical analysis

Data were analyzed by GraphPad prism 7 and presented as mean ± standard deviation (SD). *T* test was used for comparisons between two groups, and one-way analysis of variance was used for multigroup comparisons. Tukey's multiple comparisons test was used for multiple comparisons post hoc. *P* < 0.05 was considered statistically significant. Sequences of the PCR primers are shown in Table 1. See Additional file 1 for more experiment methods. Full-length gel images of the western blot experiment are shown in Additional files 2–29.

**Table 1:** Primer sequences

Name of primer	Sequences
miR-1a-3p-F	GCCGAGTGGAATGTAAAGAA
miR-1a-3p-R	TGGTGTCGTGGAGTCG
U6-F	CTCGCTTCGGCAGCACA
U6-R	AACGCTTCACGAATTTGCGT
A20-F	CTGCCAGCAGGTATATGGGAG
A20-R	GAACTGTGGGCAAAACTGGC
ADAR1-F	GAAGACTACGCGTTGGGACT
ADAR1-R	CTGGGAATCTTGGCCAGTGT
β-actin-F	TGTACCCAGGCATTGCTGAC
β-actin-R	AACGCAGCTCAGTAACAGTCC

*F* forward, *R* reverse

## Results

### A20 is underexpressed in the myocardium of VMC mice

**Fig. 1:**
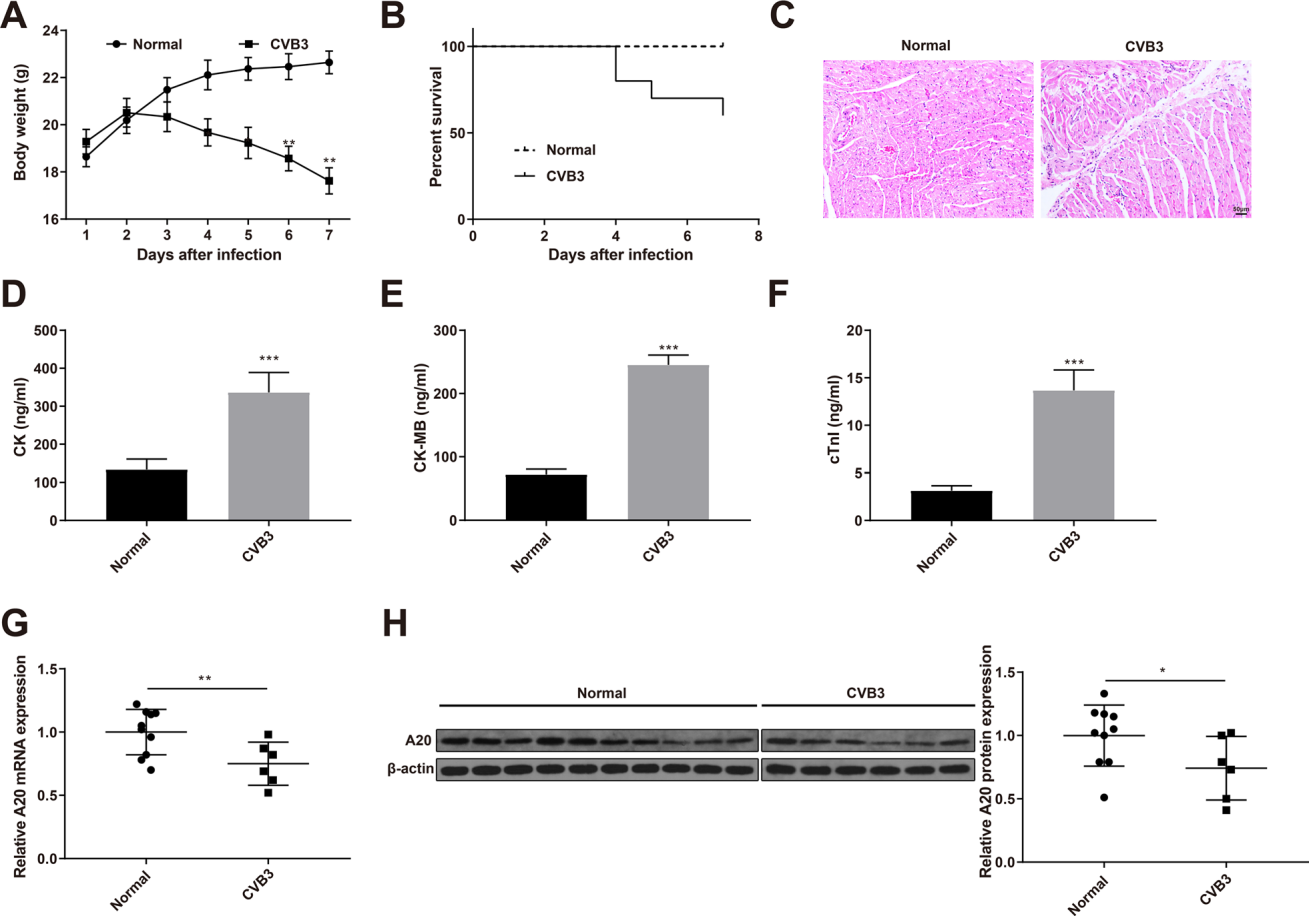
A20 is underexpressed in the myocardium of VMC mice. Notes: Male BABL/C mice were intraperitoneally injected with CVB3. Weight (**A**) and survival (**B**) of the mice; **C** H&E staining revealed the pathological changes in the myocardium; ELISA measured the serum levels of CK (**D**), CK-MB (**E**) and cTnI (**F**); qRT-PCR (**G**) and Western blotting (**H**) detected the expression of A20 in the myocardium. *P < 0.05; **P < 0.01; ***P < 0.001; *VMC* viral myocarditis, *CVB3* Coxsackie virus B3, *CK* creatine kinase, *CK-MB* creatine kinase isoenzyme, *cTnI* cardiac troponin I

The mouse weight and deaths were recorded daily after CVB3 injection. The CVB3 group continued to lose weight from day 3 (Fig. 1A) and died from day 4 (Fig. 1B), compared with the Normal group. H&E staining showed inflammatory cell infiltration and cardiomyocyte necrosis in the myocardium of the CVB3 group 7 days after infection (Fig. 1C). The serum levels of CK, CK-MB and cTnI of the CVB3 group were significantly increased after 7 days, compared with the Normal group (Fig. 1D–F, *P* < 0.001). The above measurements and histological examination demonstrated successful establishment of VMC mouse models. qRT-PCR and Western blotting detected that the expression of A20 was significantly decreased in the myocardium of the CVB3 group (Fig. 1G, H, *P* < 0.05), suggesting that A20 might be implicated in VMC.

### Overexpression of A20 suppresses inflammation and apoptosis in VMC mice

**Fig. 2:**
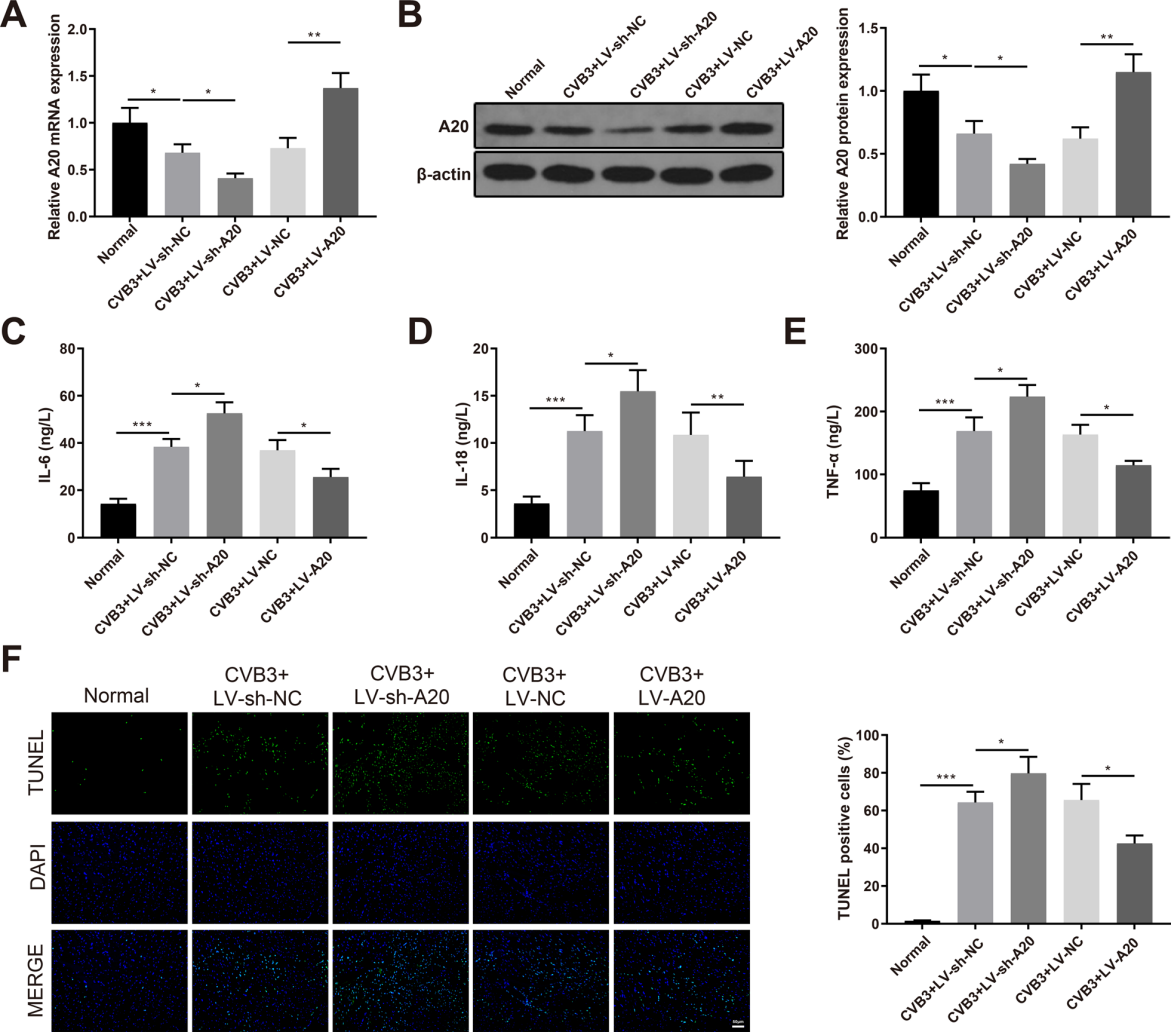
A20 suppresses inflammation and apoptosis in VMC mice. *Notes* CVB3-infected mice were injected with LV-sh-A20, LV-sh-NC, LV-A20 or LV-NC in the tail vein. qRT-PCR (**A**) and western blotting (**B**) detected the expression of A20 in the myocardium; ELISA measured the serum levels of IL-6 (**C**), IL-18 (**D**) and TNF-α (**E**); **F** TUNEL staining assessed the cell apoptosis in the myocardium. *P < 0.05; **P < 0.01; ***P < 0.001; *VMC* viral myocarditis, *CVB3* Coxsackie virus B3

CVB3-infected mice were injected with LV-sh-A20, LV-sh-NC, LV-A20 or LV-NC in the tail vein. There was no significant difference in body weight and survival rate of CVB3-infected mice in each group (data not shown). qRT-PCR and Western blotting detected the expression of A20 in the myocardium of the VMC mice. A20 was down-regulated in the CVB3 + LV-sh-A20 group and up-regulated in the CVB3 + LV-A20 group (Fig. 2A, B, *P* < 0.05, vs the CVB3 + LV-sh-NC or CVB3 + LV-NC group). ELISA showed that the serum levels of IL-6, IL-18 and TNF-α were increased in the CVB3 + LV-sh-A20 group while decreased in the CVB3 + LV-A20 group (Fig. 2C–E, *P* < 0.05, vs the CVB3 + LV-sh-NC or CVB3 + LV-NC group). TUNEL staining of the myocardial tissues showed that the cell apoptosis was increased in the CVB3 + LV-sh-A20 group while reduced in the CVB3 + LV-A20 group (Fig. 2F, *P *< 0.05, vs the CVB3 + LV-sh-NC or CVB3 + LV-NC group). Taken together, overexpression of A20 suppresses inflammation and apoptosis in VMC mice, suggesting that A20 plays a positive role in VMC.

### A20 suppresses inflammation and apoptosis of CVB3-infected cardiomyocytes

**Fig. 3:**
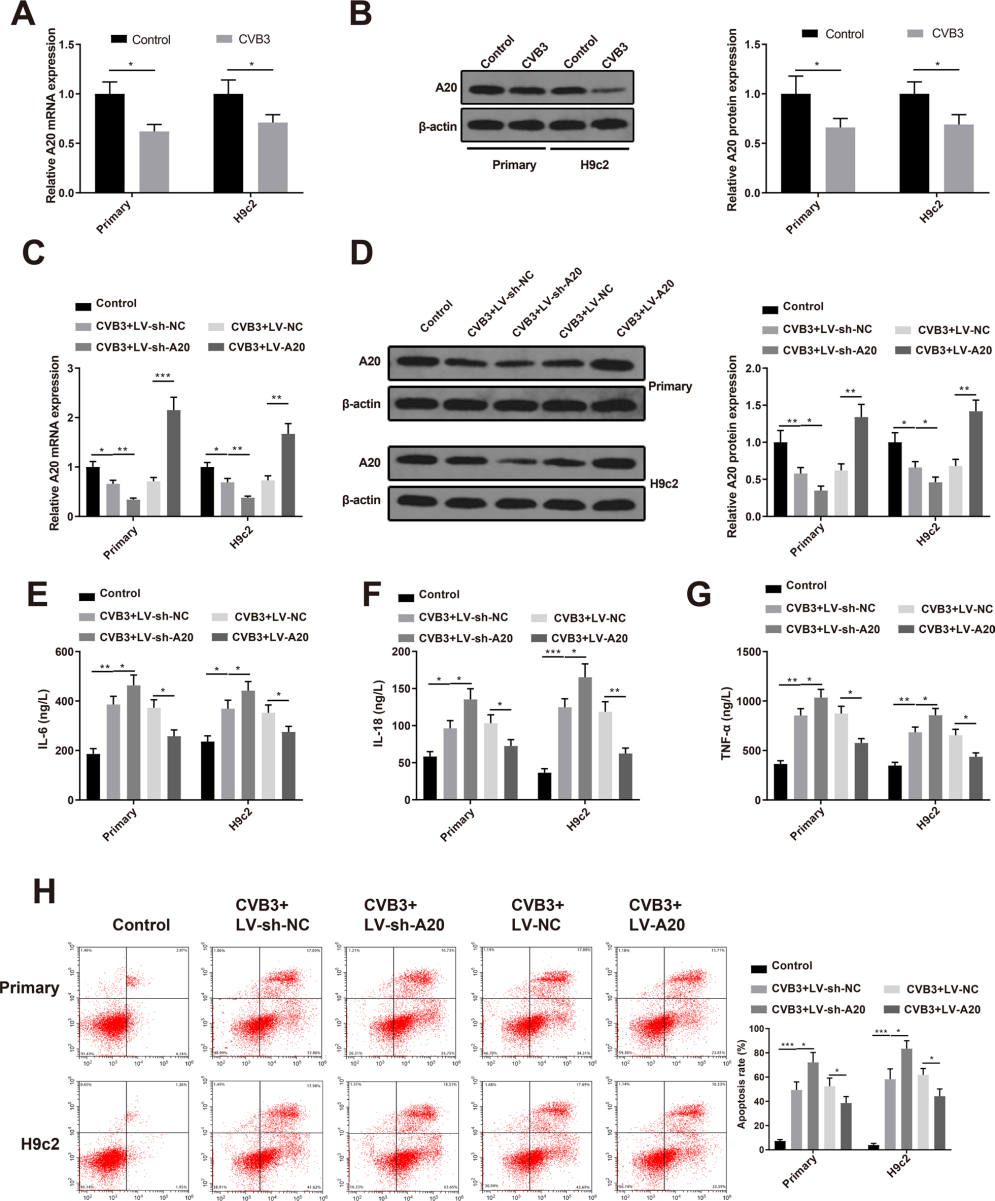
A20 suppresses inflammation and apoptosis of CVB3-infected cardiomyocytes. *Notes* qRT-PCR (**A**) and western blotting (**B**) detected the expression of A20 in primary neonatal rat cardiomyocytes and H9c2 cells. CVB3-infected primary neonatal rat cardiomyocytes and H9c2 cells were transfected with LV-sh-A20, LV-sh-NC, LV-A20 or LV-NC. qRT-PCR (**C**) and western blotting (**D**) detected the expression of A20 in the cells; ELISA measured the levels of IL-6 (**E**), IL-18 (**F**) and TNF-α (**G**) in the supernatant; **H** flow cytometry assessed the cell apoptosis. *P < 0.05; **P < 0.01; ***P < 0.001; *CVB3* Coxsackie virus B3

qRT-PCR and Western blot analyses showed that A20 was underexpressed in CVB3-infected primary neonatal rat cardiomyocytes and H9c2 cells (Fig. 3A, B, *P* < 0.05), which was consistent with the results of animal experiments. CVB3-infected primary neonatal rat cardiomyocytes and H9c2 cells were transfected with LV-sh-A20, LV-sh-NC, LV-A20 or LV-NC. The expression of A20 was decreased in the CVB3 + LV-sh-A20 group and increased in the CVB3 + LV-A20 group (Fig. 3C, D, *P* < 0.05, vs the CVB3 + LV-sh-NC or CVB3 + LV-NC group). ELISA measured the concentrations of IL-6, IL-18 and TNF-α in the cell supernatant. The levels of IL-6, IL-18 and TNF-α were elevated in the CVB3 + LV-sh-A20 group while reduced in the CVB3 + LV-A20 group (Fig. 3E–G, *P* < 0.05, vs the CVB3 + LV-sh-NC or CVB3 + LV-NC group). Flow cytometry showed that the apoptosis rate was increased in the CVB3 + LV-sh-A20 group while decreased in the CVB3 + LV-A20 group (Fig. 3H, *P* < 0.05, vs the CVB3 + LV-sh-NC or CVB3 + LV-NC group). Taken together, A20 protects primary neonatal rat cardiomyocytes and H9c2 cells against CVB3-induced inflammation and apoptosis.

### A20 is a target gene of miR-1a-3p

**Fig. 4:**
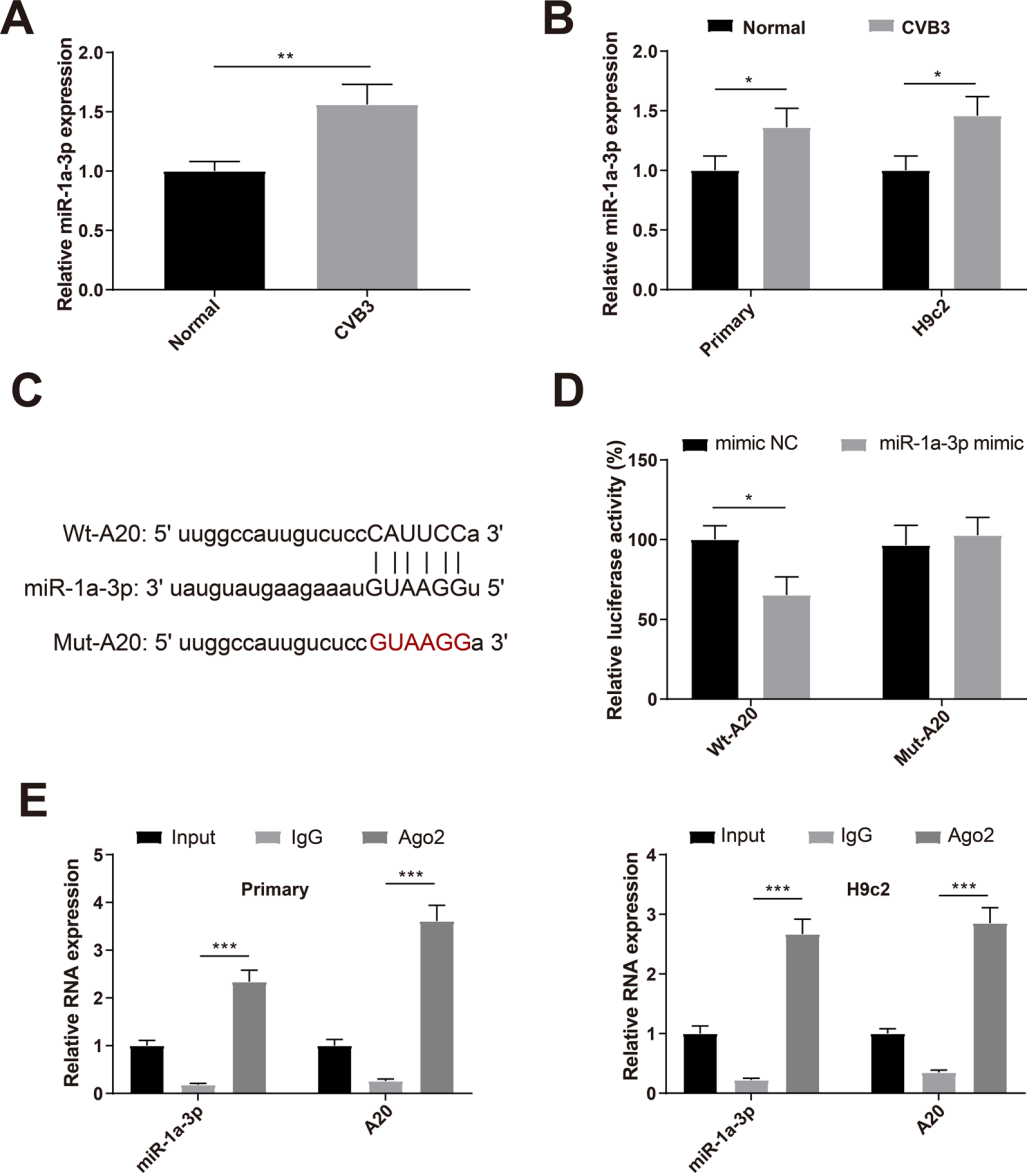
A20 is a target gene of miR-1a-3p. *Notes* qRT-PCR detected the expression of miR-1a-3p in the myocardium of VMC mice (**A**) and in CVB3-infected primary neonatal rat cardiomyocytes and H9c2 cells (**B**); **C** starBase predicted the binding sites between miR-1a-3p and A20; dual-luciferase reporter assay (**D**) and RIP assay (**E**) verified the binding between miR-1a-3p and A20. *P < 0.05; **P < 0.01; ***P < 0.001; *VMC* viral myocarditis, *CVB3* Coxsackie virus B3

qRT-PCR detected that miR-1a-3p was highly expressed in the myocardium of VMC mice (Fig. 4A , *P* < 0.01) as well as in CVB3-infected primary neonatal rat cardiomyocytes and H9c2 cells (Fig. 4B, *P* < 0.05). StarBase predicted A20-binding miRNAs, and the binding sites between A20 and miR-1a-3p are shown in Fig. 4C. Dual-luciferase reporter assay showed that the Wt-A20 + miR-1a-3p mimic group had reduced luciferase activity compared with the Wt-A20 + mimic NC group while the Mut-A20 + miR-1a-3p mimic and Mut-A20 + mimic NC groups had no significant change in the relative luciferase activity (Fig. 4D). RIP assay showed that Ago2 antibody, instead of IgG antibody, enriched endogenous A20 mRNA of primary neonatal rat cardiomyocytes or H9c2 cells (Fig. 4E, *P* < 0.001). Dual-luciferase reporter and RIP assays both validated the binding between A20 and miR-1a-3p.

### miR-1a-3p promotes inflammation and apoptosis of CVB3-infected cardiomyocytes by targeting A20

**Fig. 5:**
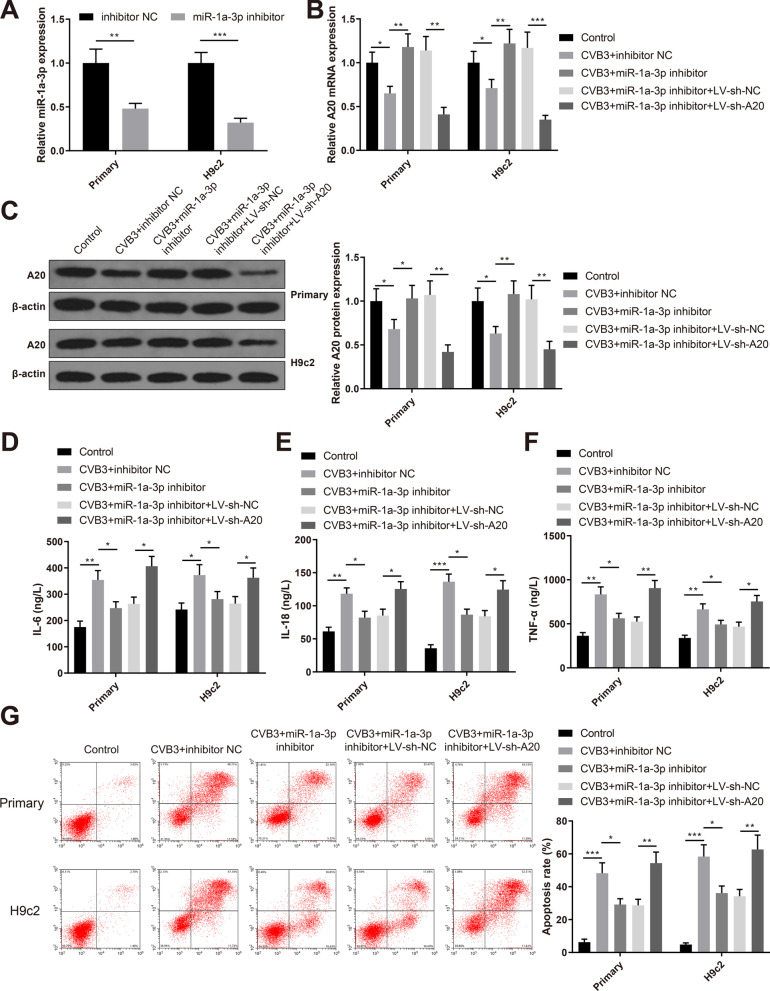
miR-1a-3p promotes inflammation and apoptosis of CVB3-infected cardiomyocytes by targeting A20. *Notes* CVB3-infected primary neonatal rat cardiomyocytes and H9c2 cells were transfected with miR-1a-3p inhibitor, inhibitor NC, miR-1a-3p inhibitor + LV-sh-NC or miR-1a-3p inhibitor + LV-sh-A20. **A** qRT-PCR detected the expression of miR-1a-3p; qRT-PCR (**B**) and western blotting (**C**) detected the expression of A20; ELISA measured the levels of IL-6 (**D**), IL-18 (**E**) and TNF-α (**F**) in the supernatant; **G** flow cytometry assessed the cell apoptosis. *P < 0.05; **P < 0.01; ***P < 0.001; *CVB3* Coxsackie virus B3

To determine the regulation of miR-1a-3p on A20 in VMC, we transfected CVB3-infected primary neonatal rat cardiomyocytes and H9c2 cells with miR-1a-3p inhibitor, inhibitor NC, miR-1a-3p inhibitor + LV-sh-NC or miR-1a-3p inhibitor + LV-sh-A20. The expression of miR-1a-3p was decreased in the CVB3 + miR-1a-3p inhibitor group compared with the CVB3 + inhibitor NC group (Fig. 5A, *P* < 0.01). Moreover, the expression of A20 was elevated in the CVB3 + miR-1a-3p inhibitor group while down-regulated in the CVB3 + miR-1a-3p inhibitor + LV-sh-A20 group (Fig. 5B, C, *P* < 0.05, vs the CVB3 + inhibitor NC or CVB3 + miR-1a-3p inhibitor + LV-sh-NC group). ELISA showed that the levels of IL-6, IL-18 and TNF-α were decreased in the CVB3 + miR-1a-3p inhibitor group while increased in the CVB3 + miR-1a-3p inhibitor + LV-sh-A20 group (Fig. 5D–F, *P* < 0.05, vs the CVB3 + inhibitor NC or CVB3 + miR-1a-3p inhibitor + LV-sh-NC group). Flow cytometry showed that the apoptosis rate was also decreased in the CVB3 + miR-1a-3p inhibitor group while increased in the CVB3 + miR-1a-3p inhibitor + LV-sh-A20 group (Fig. 5G, *P* < 0.05, vs the CVB3 + inhibitor NC or CVB3 + miR-1a-3p inhibitor + LV-sh-NC group). Taken together, miR-1a-3p promotes the inflammation and apoptosis of CVB3-infected cardiomyocytes by targeting A20.

### ADAR1 promotes the expression of miR-1a-3p by binding to Dicer

**Fig. 6:**
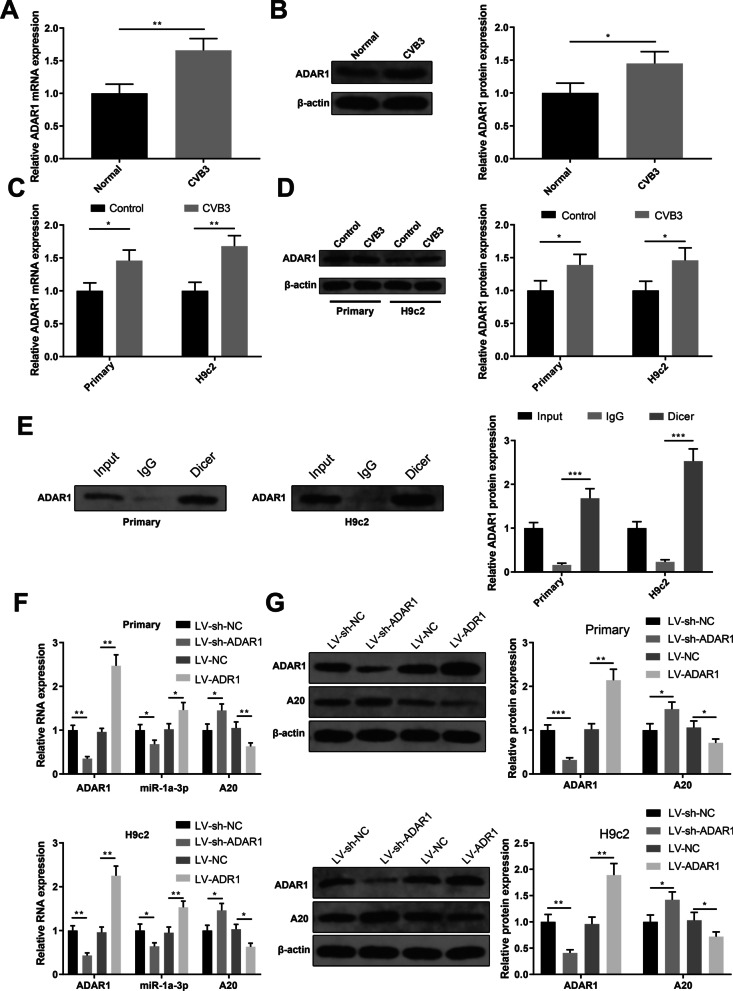
ADAR1 promotes the expression of miR-1a-3p by binding to Dicer. *Notes* qRT-PCR and Western blotting detected the expression of ADAR1 in the myocardium of VMC mice (**A**, **B**), and in CVB3-infected primary neonatal rat cardiomyocytes and H9c2 cells (**C**, **D**); **E** Co-IP assay verified the binding between ADAR1 and Dicer; qRT-PCR (**F**) and Western blotting (**G**) detected the expressions of ADAR1, miR-1a-3p and A20 in primary neonatal rat cardiomyocytes and H9c2 cells that were transfected with LV-sh-NC, LV-sh-ADAR1, LV-NC or LV-ADAR1. *P < 0.05; **P < 0.01; ***P < 0.001; *VMC* viral myocarditis, *CVB3* Coxsackie virus B3, *Co-IP* co-immunoprecipitation

The mechanism regarding the upregulation of miR-1a-3p in VMC was further investigated. qRT-PCR and Western blotting detected that ADAR1 was highly expressed in the myocardium of VMC mice (Fig. 6A, B, *P* < 0.05) as well as in CVB3-infected primary neonatal rat cardiomyocytes and H9c2 cells (Fig. 6C, D, *P* < 0.05). RPISeq (http://pridb.gdcb.iastate.edu/RPISeq/) analysis of the relationship between ADAR1 and miR-1a-3p precursor (pre-miR-1a-3p) showed that the interaction probabilities of these two genes were positive by two different algorithms (> 0.5). Co-IP assay was applied to verify the binding of ADAR1 to Dicer, and the results showed that Dicer antibody significantly enriched ADAR1 protein (Fig. 6E, *P* < 0.001). To further determine the regulation of ADAR1 on miR-1a-3p, we transfected primary neonatal rat cardiomyocytes and H9c2 cells with LV-sh-NC, LV-sh-ADAR1, LV-NC or LV-ADAR1. qRT-PCR and Western blot analyses showed that ADAR1 and miR-1a-3p were down-regulated while A20 was up-regulated in the LV-sh-ADAR1 group compared with the LV-sh-NC group (Fig. 6F, G, *P* < 0.05). In contrast, ADAR1 and miR-1a-3p were up-regulated while A20 was down-regulated in the LV-ADAR1 group compared with the LV-NC group (Fig. 6F, G, *P* < 0.05). Altogether, ADAR1 mediates the expressions of miR-1a-3p/A20 by binding to Dicer.

## Discussion

VMC is an inflammatory disease caused by viral infections which induce host immune response and eventually lead to cardiomyocyte dysfunction and compromised contractility. The molecular regulation in the progression of VMC remains an elusive problem for understanding the pathogenesis of this disease. CVB3 was used to induce animal and cellular models to study the pathogenic mechanism of VMC. A20 was down-regulated in VMC myocardium, and A20 overexpression suppressed cardiomyocyte inflammation and apoptosis. A20 was a downstream target of miR-1a-3p, the expression of which was promoted by the ADAR1/Dicer complex. The ADAR1/miR-1a-3p/A20 axis presents important regulation in the pathogenesis of VMC.

A20 reduced the inflammation of CVB3-induced cardiomyocytes by decreasing the levels of IL-6, IL-18 and TNF-α. As one of the key regulators of NF-κB signaling, A20 plays a critical role in inflammatory and immune responses. A20 possesses a deubiquitinating motif and a zinc finger (ZF4) that act in concert to restrict the ubiquitination of receptor interacting protein 1 (RIP1), thereby inhibiting TNF-induced NF-κB signaling [16]. A20 suppresses linear ubiquitin chain assembly complex (LUBAC)-mediated NF-κB activation by binding to linear polyubiquitin via ZF7 [17]. Moreover, A20 can restrict the activation of NF-κB signaling via Toll-like receptor (TLR) or nucleotide-binding oligomerization domain containing 2 (NOD2) pathway through the disruption of ubiquitin enzyme complexes [18, 19]. Apart from the regulation of immunity, A20 also improved the cardiomyocyte survival following CVB3 infections. A20 is recruited to TNF receptor signaling complex and suppresses cell death either by stabilizing the linear ubiquitin network or by deploying deubiquitylase activities [20]. Although A20 has been demonstrated to inhibit apoptosis, its antiapoptotic effect seems to depend on the cellular context. For instance, A20 promoted TNF-induced apoptosis in intestinal epithelial cells by increasing RIPK1 activity [21]. A20 protected cardiomyocytes from CVB3-induced myocarditis, but little was known about how it was regulated in VMC.

miR-1a-3p was overexpressed in the myocardium of VMC mice as well as in CVB3-infected cardiomyocytes. A20 was demonstrated to be a downstream target of miR-1a-3p. The regulation of miR-1a-3p on A20 was later investigated in VMC cellular models. Inhibition of miR-1a-3p suppressed CVB3-induced inflammation and apoptosis of cardiomyocytes, whereas A20 knockdown reversed the suppressive effects of miR-1a-3p inhibitor. Altogether, miR-1a-3p promoted the inflammation and apoptosis of CVB3-induced cardiomyocytes by targeting A20. Existing evidence indicates that miR-1a-3p is a myogenesis- and apoptosis-associated miRNA. miR-1a-3p collaborated with miR-133a-3p on myoblast differentiation and skeletal muscle growth through activation of the AKT/mTOR/S6K signaling pathway [22]. Omega-3 polyunsaturated fatty acid supported cardiac healing following myocardial infarction in fat-1 transgenic mice by regulating several apoptosis-associated miRNAs including miR-1a-3p [23]. Soluble epoxide hydrolase inhibitors prevented ischemic arrhythmias by repressing the expression of miR-1 [24]. miR-1a-3p was up-regulated in response to infectious bronchitis virus infections in chicken kidneys [25]. However, miR-1a-3p not always plays a negative role. miR-1a-3p attenuated isoproterenol-induced heart failure by increasing the expressions of mitochondrial ND1 and COX1 [26]. miR-1a-3p was required for the maintenance of heart histology and function in cardiac-selective miRNA deficient mice [27].

Furthermore, we found that ADAR1 was also overexpressed in the myocardium of VMC mice as well as in CVB3-induced cardiomyocytes. ADAR1 prevents autoimmunity during viral infection by blocking canonical antiviral pathways including RIG-I-like receptor, PKR, and oligoadenylate synthetase-RNase L [28]. Studies have demonstrated that ADAR1 is implicated in the replication of various viruses such as Zika virus [29], hepatitis B virus [30] and human papillomavirus [31]. ADAR1 functions as a mammalian RNA-editing enzyme that converts selected adenosine residuals to inosine in the double-stranded regions of RNA transcripts (dsRNA) [32]. More recently, ADAR1 has been found to promote miRNA processing by forming a complex with Dicer [33]. The enzyme Dicer is a well-characterized endonuclease that cleaves long dsRNA molecules into small RNAs, contributing to the formation of RNA-induced silencing complex [34]. In the present study, ADAR1 upregulated the expression of miR-1a-3p while downregulated the expression of A20 in cardiomyocytes. Also, ADAR1 was demonstrated to bind with Dicer in cardiomyocytes. Altogether, ADAR1 promoted the cleavage of pre-miR-1a-3p by binding to Dicer, thereby regulating the functional activities of A20.

A20, regulated by the ADAR1/miR-1a-3p axis, suppresses cardiomyocyte inflammation and apoptosis in CVB3-induced myocarditis. Many studies have suggested A20 as a drug-able target in immunotherapy. This study not only uncovers the effect but also the regulators of A20 in VMC, providing insights into the complete regulatory mechanisms associated with A20. Investigation into the headstreams of A20 may be of great value for mitigating A20 deficiency-induced inflammatory diseases.

## Supplementary Information

**Additional file 1.** More detailed experimental methods.

**Additional file 2.** Full-length gel images of fig1H-CVB3-A20.

**Additional file 3.** Full-length gel images of fig1H-Normal-A20.

**Additional file 4.** Full-length gel images of fig1H-CVB3-β-actin.

**Additional file 5.** Full-length gel images of fig1H-Normal-β-actin.

**Additional file 6.** Full-length gel images of fig2B-A20.

**Additional file 7.** Full-length gel images of fig2B-β-actin.

**Additional file 8.** Full-length gel images of fig3B-A20.

**Additional file 9.** Full-length gel images of fig3B-β-actin.

**Additional file 10.** Full-length gel images of fig3D-A20-H9c2.

**Additional file 11.** Full-length gel images of fig3D-A20-primary.

**Additional file 12.** Full-length gel images of fig3D-β-actin-H9c2.

**Additional file 13.** Full-length gel images of fig3D-β-actin-H9c2.

**Additional file 14.** Full-length gel images of fig5C-A20-H9c2.

**Additional file 15.** Full-length gel images of fig5C-A20-primary.

**Additional file 16.** Full-length gel images of fig5C-β-actin-H9c2.

**Additional file 17.** Full-length gel images of fig5C-β-actin-H9c2.

**Additional file 18.** Full-length gel images of fig6B-ADAR1.

**Additional file 19.** Full-length gel images of fig6B-β-actin.

**Additional file 20.** Full-length gel images of fig6D-ADAR1.

**Additional file 21.** Full-length gel images of fig6D-β-actin.

**Additional file 22.** Full-length gel images of fig6E-ADAR1-H9c2.

**Additional file 23.** Full-length gel images of fig6E-ADAR1-primary.

**Additional file 24.** Full-length gel images of fig6G-A20-H9c2.

**Additional file 25.** Full-length gel images of fig6G-A20-primary.

**Additional file 26.** Full-length gel images of fig6G-ADAR1-H9c2.

**Additional file 27.** Full-length gel images of fig6G-ADAR1-primary.

**Additional file 28.** Full-length gel images of fig6G-β-actin-H9c2.

**Additional file 29.** Full-length gel images of fig6G-β-actin-primary.

## Data Availability

The datasets used or analyzed during the current study are available from the corresponding author on reasonable request.
