# Supplementary material for: A20 (TNFAIP3) alleviates viral myocarditis through ADAR1/miR-1a-3p-dependent regulation
Source: BMC Cardiovasc Disord. 2022 Jan 16;22:10. doi: 10.1186/s12872-021-02438-z (PMC8762865; doi:10.1186/s12872-021-02438-z)
Supplement: Supplementary file 1 — Additional file 1. More detailed experimental methods. [file 12872_2021_2438_MOESM1_ESM.docx]

**H&E staining**

Myocardial tissues of the mice were fixed in 4% formaldehyde solution for 24 h and made into 4-μm paraffin sections. The sections were dewaxed in xylene and hydrated in gradient alcohol. The tissues were stained in hematoxylin for 5 min. Excessive staining solution was washed off, and the tissues were treated with hydrochloric acid-ethanol for 30 s followed by eosin staining for 2 min. The tissues were routinely dehydrated, transparentized and mounted. The tissues were observed and photographed under a microscope.

**ELISA**

ELISA kits (R&D, USA) were used to measure the serum levels of creatine kinase (CK), creatine kinase isoenzyme (CK-MB) and cardiac troponin I (cTnI), and the levels of IL-6, IL-18 and TNF-α in the serum and cell culture supernatant. All operations strictly followed the instructions of the kits.

**qRT-PCR**

TRIzol (Invitrogen, Carlsbad, CA, USA) was used for extraction of total RNA from tissues or cells. Reverse transcription of RNA was performed using reverse transcription kit (TaKaRa, Tokyo, Japan) following the instruction. Gene expression was detected by LightCycler 480 (Roche, Indianapolis, IN, USA), and the reaction conditions were set according to the instruction of SYBR Green Mix (Roche Diagnostics, Indianapolis, IN). The thermal cycling parameters were as follows: 10 s at 95°C; 45 cycles of 5 s at 95°C, 10 s at 60°C and 10 s at 72°C; 5 min at 72°C. Each reaction was performed in triplicate. U6 and β-actin were the respective internal references of miRNA and mRNA. Data were analyzed by the 2^-ΔΔCt^ method. ΔΔCt = experimental group (Ct target gene-Ct reference gene)-control group (Ct target gene-Ct reference gene). Sequences of the amplification primer of each gene are shown in Table 1.

**Western blotting**

Tissues or cells were lysed in RIPA lysis buffer (Beyotime, Shanghai, China). Protein was extracted from the tissues or cells, and quantified by BCA kit (Beyotime). Corresponding volume of proteins were added into loading buffer (Beyotime) and heated in boiling water for 3 min. The electrophoresis was run at 80 V for 30 min, and continued at 120 V for 1 ~ 2 h after bromophenol blue came into separation gel. The proteins were transferred onto a membrane in an ice bath at 300 mA for 60 min. The membrane was rinsed in washing buffer for 1 ~ 2 min, and then soaked in blocking buffer at room temperature for 60 min or at 4°C overnight. The proteins were incubated with primary antibodies against β-actin (4970S, 1:1000), A20 (5630S, 1:1000) and ADAR1 (14175S, 1:10000) (Cell Signaling, Boston, USA) on a shaking bed at room temperature for 1 h. The membrane was rinsed in washing buffer for 3 × 10 min and then transferred into secondary antibody solution at room temperature for 1 h followed by another round of washing. The membrane was added with developing solution, and the protein expression was detected by a chemiluminescence imaging system (Gel Doc XR, Bio-rad).

**TUNEL staining**

Paraffin sections of myocardial tissue were rinsed in xylene for 5 min, and in 100%, 95%, 90%, 80% and 70% alcohol solution for 3 min followed by two PBS washes. The myocardial tissues were treated with proteinase K for 30 min and washed twice with PBS. Then, the tissues were soaked in blocking buffer for 10 min and washed twice with PBS. The tissues were added with premixed solution of TdT (2 μl) and red florescent Cy3 probe-labelled dUTP (48 μl) (Beyotime, Shanghai, China), and placed in a dark and wet box for 1 h. Following three PBS washes, the tissues were mounted with anti-fluorescence quenching sealing solution and observed under a fluorescence microscope. Cell nuclei were stained by DAPI. TUNEL positive cells (%) = red cell number/total cell number × 100%.

**Flow cytometry**

Suspension of transfected cells (3ml, 1 × 10^5^ cells/ml) was centrifuged in 10-ml centrifuge tubes at 500 r/min for 5 min. The culture solution was discarded after the centrifugation. Following PBS wash, the cells were centrifuged at 500 r/min for 5 min, and the supernatant was then discarded. The cells were resuspended in 100 μl of binding buffer, and added with 5 μl of Annexin V-FITC and 5 μl of PI. The reaction lasted for 15 min at room temperature in the dark. FITC and PI fluorescence was detected by a flow cytometer for apoptosis analysis. The assay was repeated for three times.

**RNA-binding protein immunoprecipitation (RIP) assay**

Magna RIP™ kit (Millipore, Billerica, MA, USA) was used for the RIP assay. Primary neonatal rat cardiomyocytes or H9c2 cells were washed twice with pre-cooled PBS, centrifuged at 1,500 rpm for 5 min, and added with equal volume of RIP lysis buffer. Beads were resuspended in 100 μl of RIP wash buffer, and added with 5 μg of Ago2 antibodies (ab32381, 1:100, Abcam, Cambridge, MA, USA) or IgG antibodies. The antibodies were incubated with the beads at room temperature for 30 min. The centrifuge tube was placed onto a magnetic grate, and supernatant in the tube was discarded. RIP wash buffer (500 μl) was added into the tube, and supernatant was discarded after vortex oscillation. The aforementioned step was repeated once. The tube was added with 500 μl of RIP wash buffer, and placed on ice after vortex oscillation. The bead tube was placed onto the magnetic grate, and supernatant in the tube was discarded. Each bead tube was added with 900 μl of RIP immunoprecipitation buffer. The cell lysates were thawed and centrifuged at 14,000 rpm, 4°C for 10 min. Supernatant of the cell lysates (100 μl) was added into the bead-antibody complexes, incubated at 4°C overnight, and then centrifuged for a short while. The centrifuge tube was placed onto the magnetic grate, and supernatant in the tube was discarded. The tube was added with 500 μl of RIP wash buffer, and placed onto the magnetic grate after vortex oscillation to discard the supernatant. The bead-antibody complexes were washed for six times as stated. The complexes were resuspended in 150 μl of proteinase K buffer, and incubated at 55°C for 30 min. The tube was placed on the magnetic grate, and supernatant in the tube was collected for qRT-PCR detection of the mRNA levels of miR-1a-3p and A20.

**Dual-luciferase reporter assay**

The binding sites between miR-1a-3p and A20 were predicted by starBase (http://starbase.sysu.edu.cn/). Wild and mutated sequences of the binding site on A20 (Wt-A20 and Mut-A20) were designed and synthesized according to the prediction. Wt-A20 or Mut-A20 was inserted into pGL3-Basic vectors (Promega, Madison, WI, USA), and then co-transfected with miR-1a-3p mimic or mimic NC (50 nM) into HEK293T cells (ATCC, Manassas, Virginia, USA). pRL-TK vectors were used as the internal control and co-transfected with the pGL3-Basic vectors into HEK293T cells. Activity of the Firefly and Renilla luciferases was detected by a dual-luciferase reporter assay kit (Promega, Madison, WI, USA). Renilla luciferase activity was used as the internal control, and the ratio of Firefly luciferase activity to Renilla luciferase activity was the relative luciferase activity.

**Co-immunoprecipitation (co-IP) assay**

Primary neonatal rat cardiomyocytes or H9c2 cells were added with RIPA lysis buffer, and then centrifuged at 14,000 g, 4°C for 15 min. Supernatant was transferred to a new centrifuge tube, and added with diluted Dicer antibodies (sc-136979, 1 μg, Santa Cruz Biotechnology, Dallas, TX, USA). The tube was placed onto a shaking bed at 4°C overnight or gently shaken at room temperature for 2 h. The antigen-antibody complexes were incubated with protein A/G agarose bead solution (100 μl; bead: PBS = 1:1) in the shaking bed at 4°C overnight or at room temperature for 1 h. The mixture was centrifuged at 14,000 rpm for 5 s, and supernatant was discarded. The bead-antigen-antibody complexes were rinsed with 800 μl of pre-cooled RIPA buffer for three times, and suspended in 60 μl of 2 × loading buffer. The loading samples were boiled for 5 min and centrifuged at 14,000 g. Supernatant was boiled for 5 min before electrophoresis and Western blotting.
