# Supplementary figures and images for: A20 (TNFAIP3) alleviates viral myocarditis through ADAR1/miR-1a-3p-dependent regulation
Source: BMC Cardiovasc Disord. 2022 Jan 16;22:10. doi: 10.1186/s12872-021-02438-z (PMC8762865; doi:10.1186/s12872-021-02438-z)

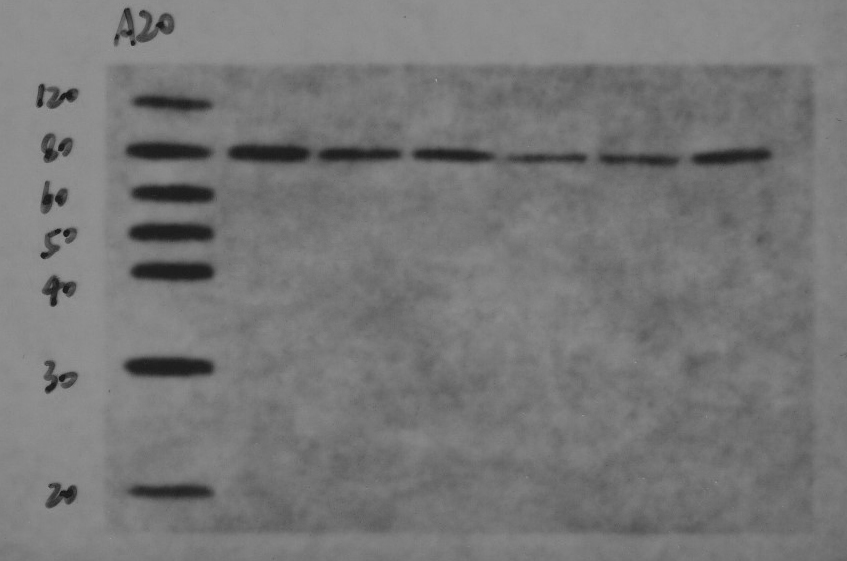

Supplement: Supplementary file 2 — Additional file 2. Full-length gel images of fig1H-CVB3-A20. [file 12872_2021_2438_MOESM2_ESM.jpg]

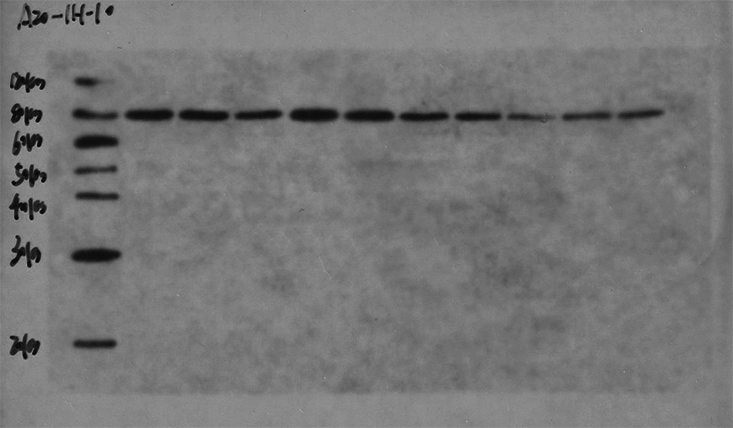

Supplement: Supplementary file 3 — Additional file 3. Full-length gel images of fig1H-Normal-A20. [file 12872_2021_2438_MOESM3_ESM.jpg]

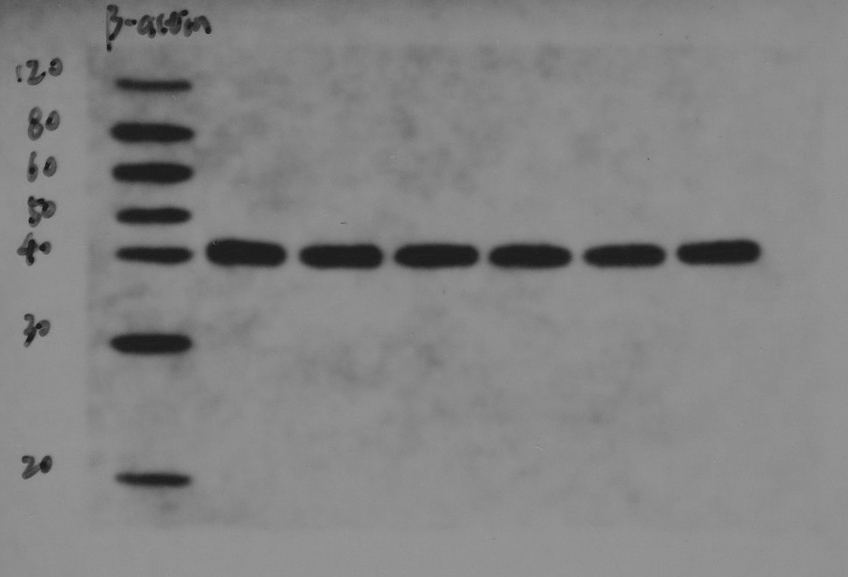

Supplement: Supplementary file 4 — Additional file 4. Full-length gel images of fig1H-CVB3-β-actin. [file 12872_2021_2438_MOESM4_ESM.jpg]

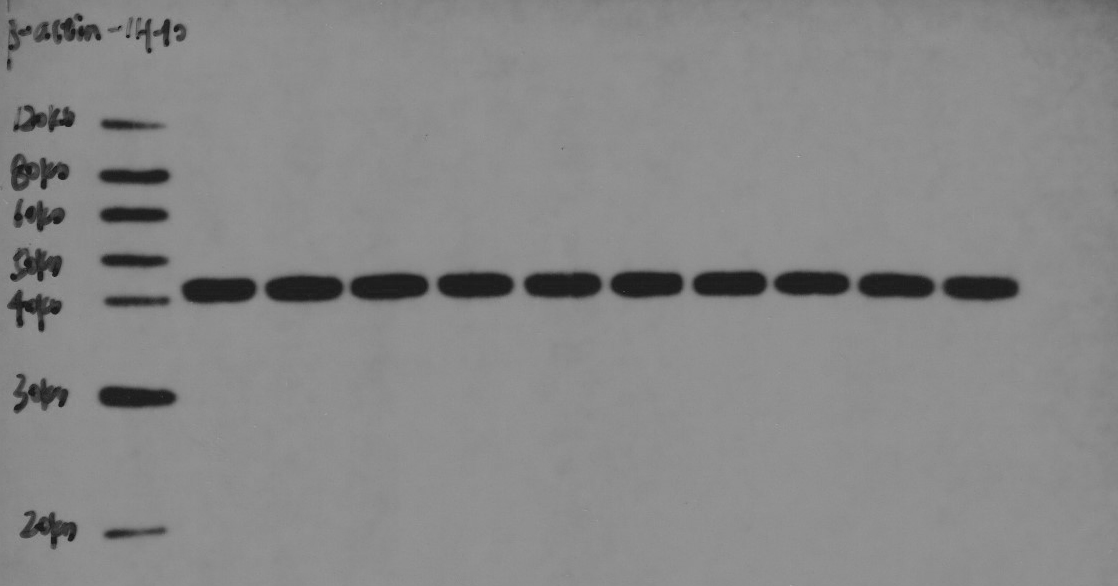

Supplement: Supplementary file 5 — Additional file 5. Full-length gel images of fig1H-Normal-β-actin. [file 12872_2021_2438_MOESM5_ESM.jpg]

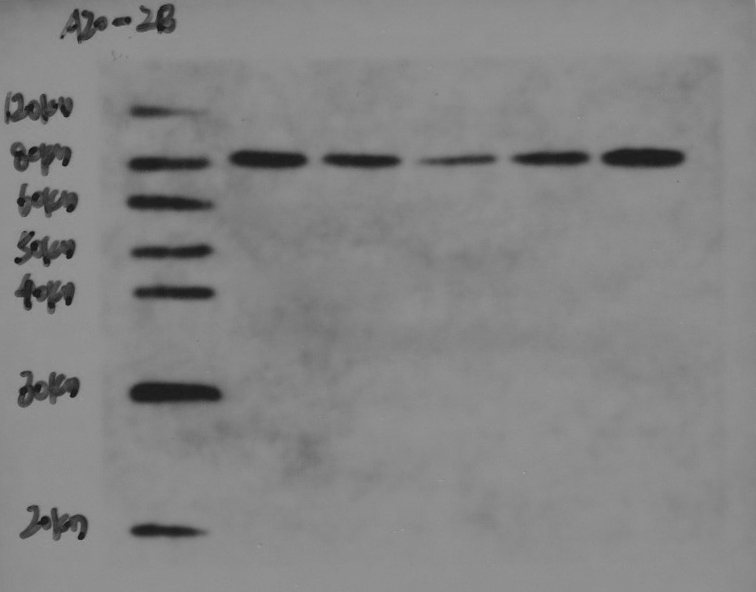

Supplement: Supplementary file 6 — Additional file 6. Full-length gel images of fig2B-A20. [file 12872_2021_2438_MOESM6_ESM.jpg]

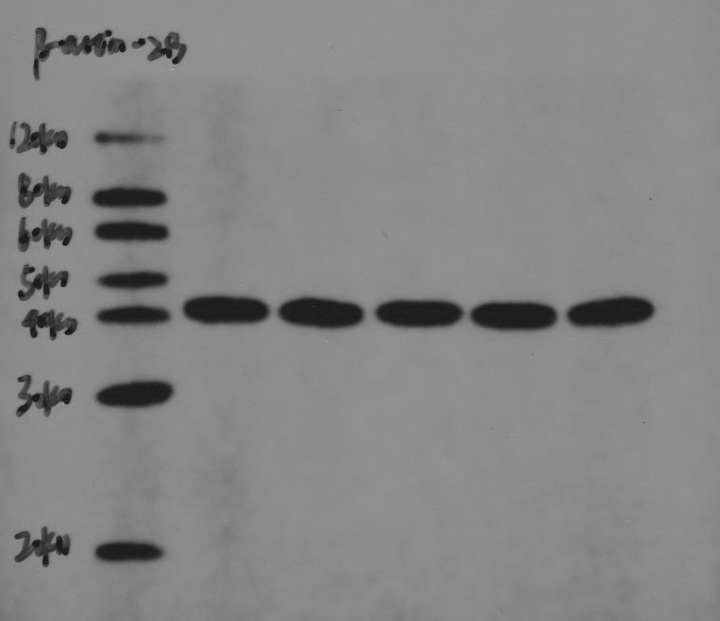

Supplement: Supplementary file 7 — Additional file 7. Full-length gel images of fig2B-β-actin. [file 12872_2021_2438_MOESM7_ESM.jpg]

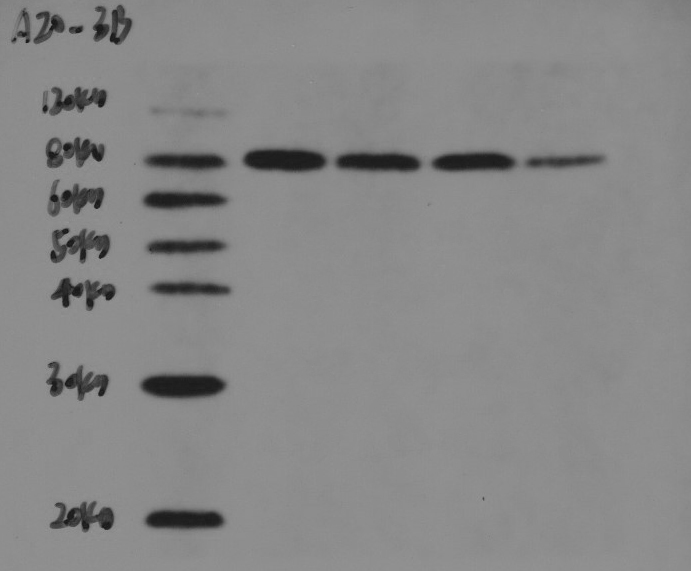

Supplement: Supplementary file 8 — Additional file 8. Full-length gel images of fig3B-A20. [file 12872_2021_2438_MOESM8_ESM.jpg]

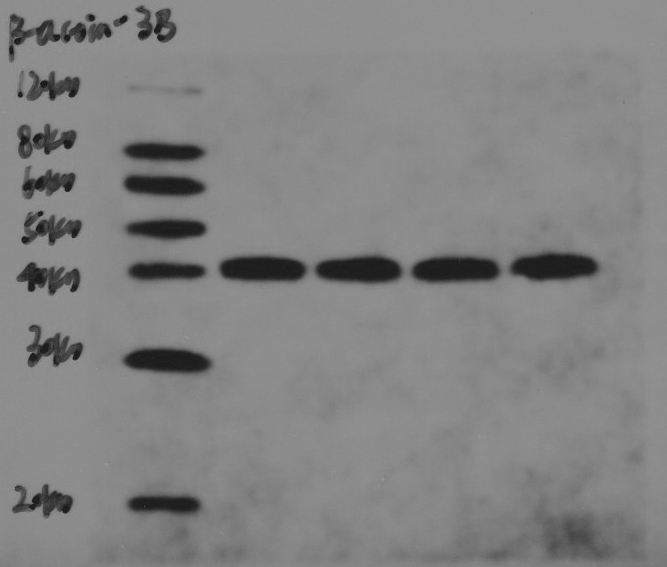

Supplement: Supplementary file 9 — Additional file 9. Full-length gel images of fig3B-β-actin. [file 12872_2021_2438_MOESM9_ESM.jpg]

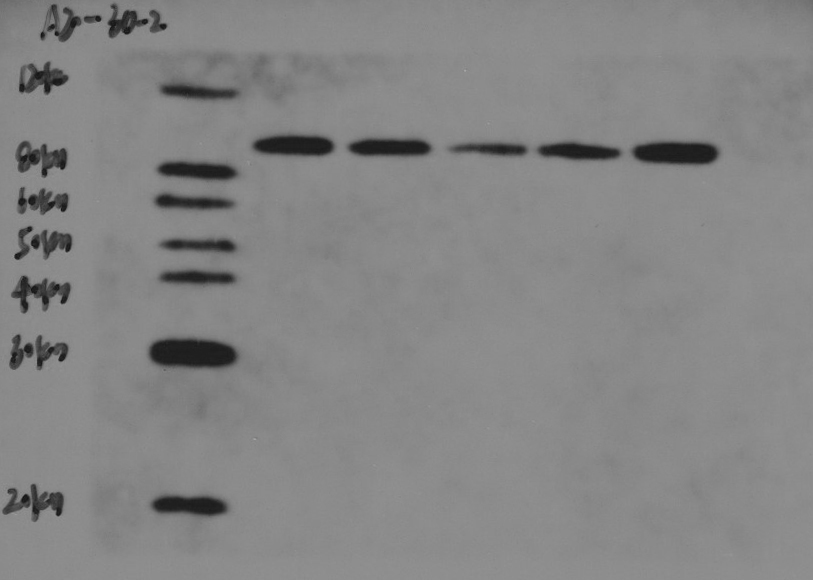

Supplement: Supplementary file 10 — Additional file 10. Full-length gel images of fig3D-A20-H9c2. [file 12872_2021_2438_MOESM10_ESM.jpg]

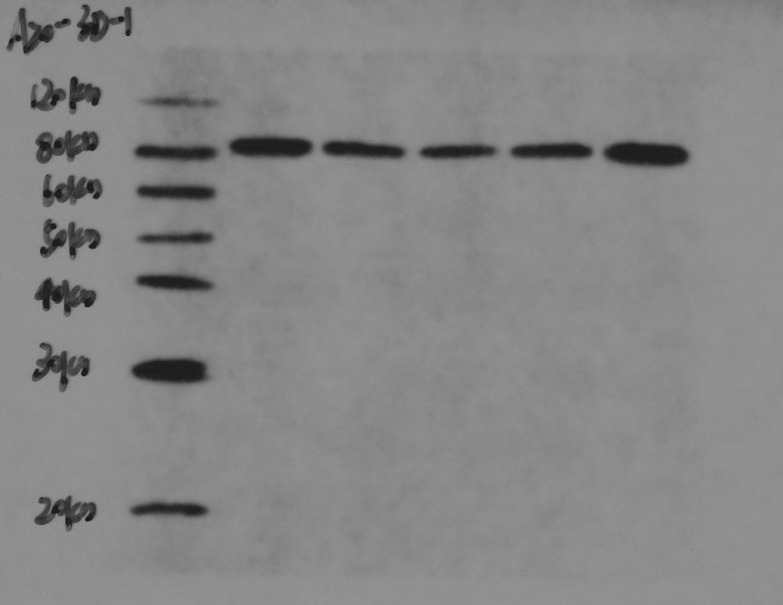

Supplement: Supplementary file 11 — Additional file 11. Full-length gel images of fig3D-A20-primary. [file 12872_2021_2438_MOESM11_ESM.jpg]

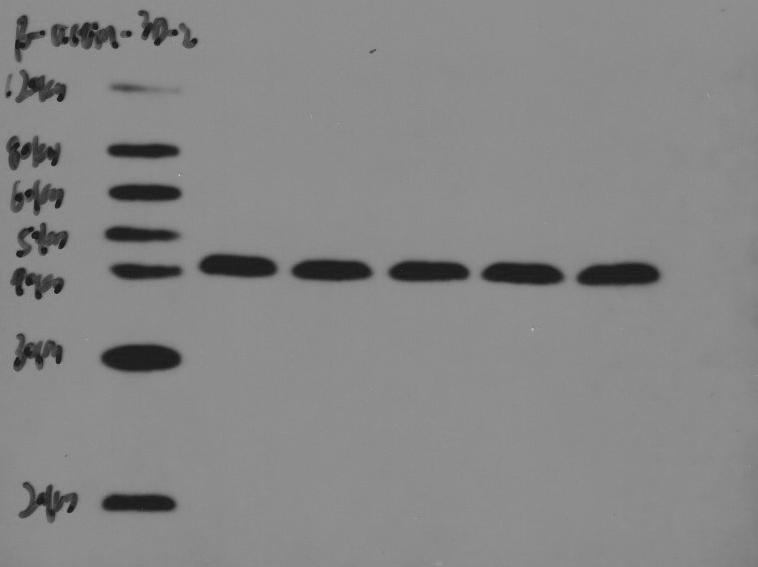

Supplement: Supplementary file 12 — Additional file 12. Full-length gel images of fig3D-β-actin-H9c2. [file 12872_2021_2438_MOESM12_ESM.jpg]

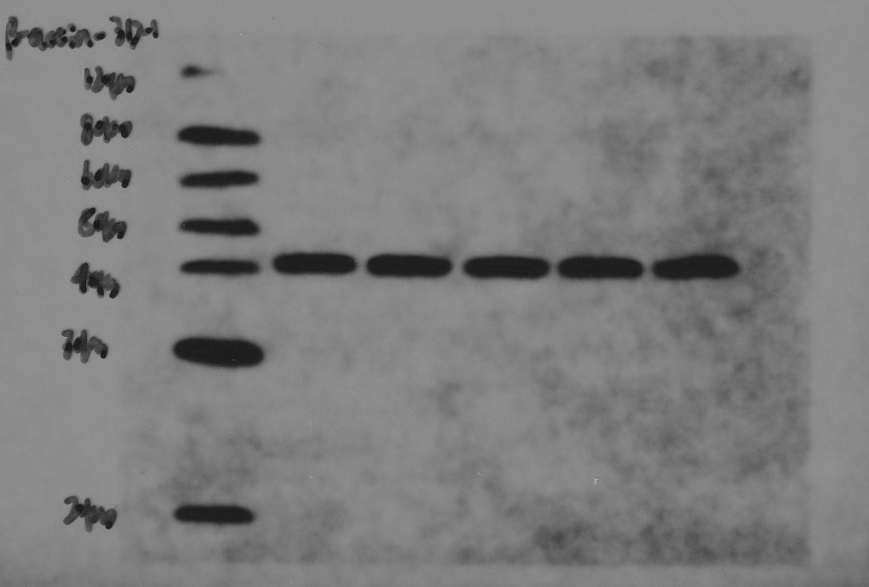

Supplement: Supplementary file 13 — Additional file 13. Full-length gel images of fig3D-β-actin-H9c2. [file 12872_2021_2438_MOESM13_ESM.jpg]

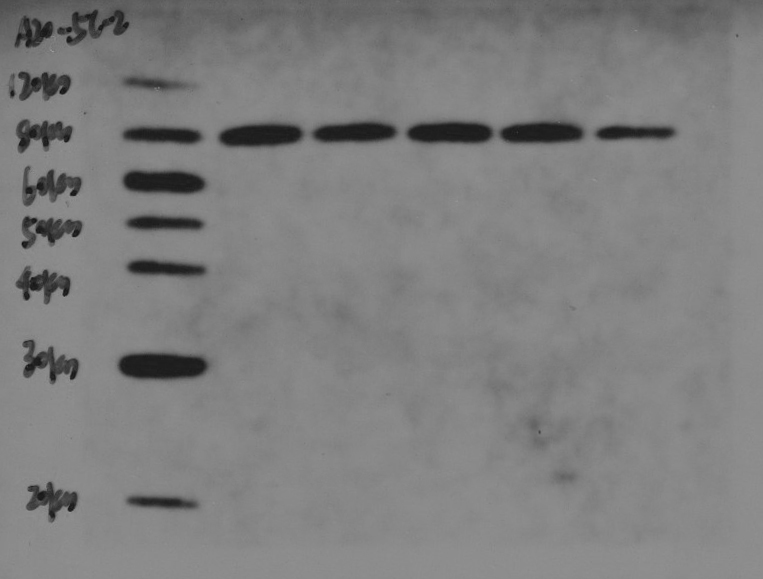

Supplement: Supplementary file 14 — Additional file 14. Full-length gel images of fig5C-A20-H9c2. [file 12872_2021_2438_MOESM14_ESM.jpg]

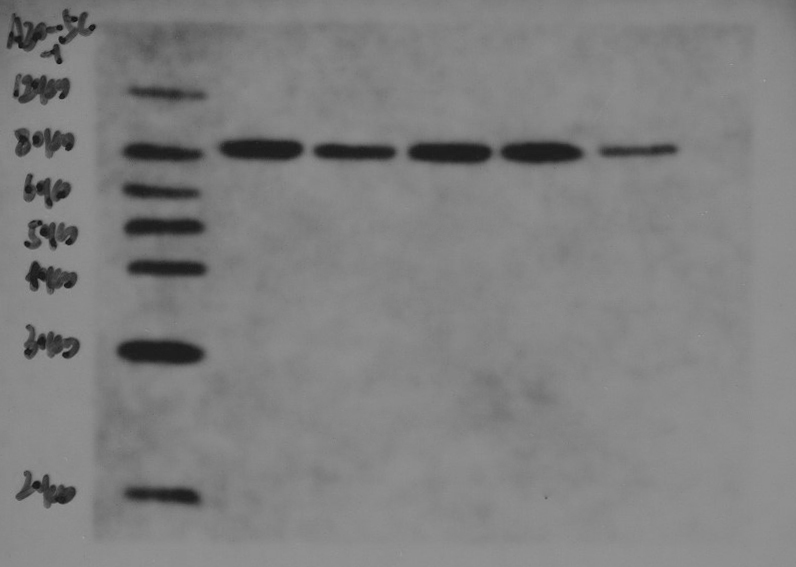

Supplement: Supplementary file 15 — Additional file 15. Full-length gel images of fig5C-A20-primary. [file 12872_2021_2438_MOESM15_ESM.jpg]

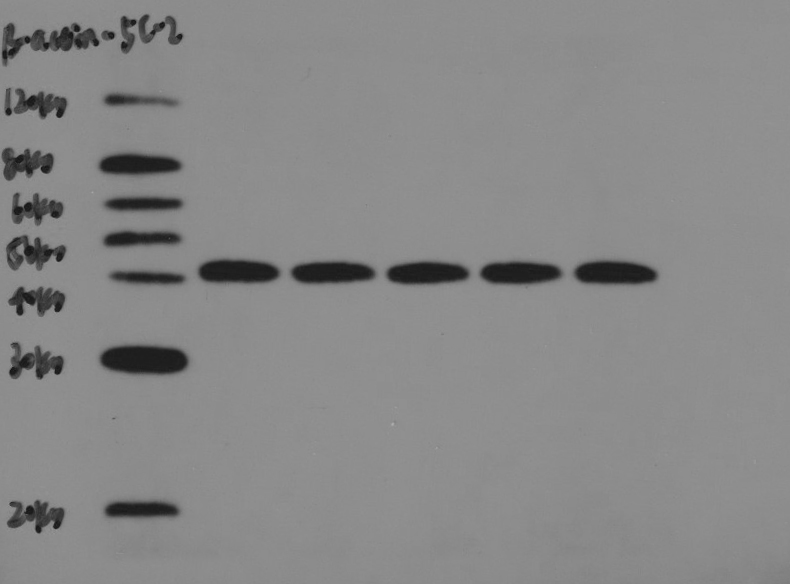

Supplement: Supplementary file 16 — Additional file 16. Full-length gel images of fig5C-β-actin-H9c2. [file 12872_2021_2438_MOESM16_ESM.jpg]

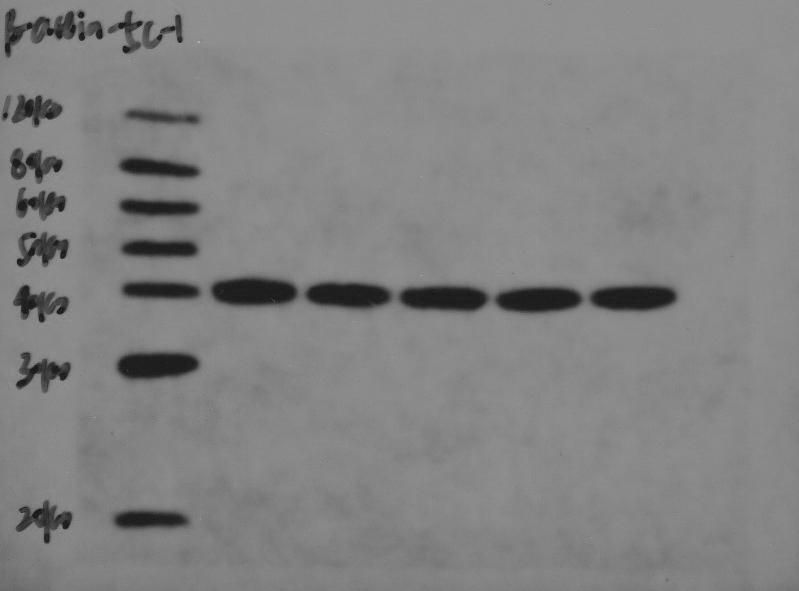

Supplement: Supplementary file 17 — Additional file 17. Full-length gel images of fig5C-β-actin-H9c2. [file 12872_2021_2438_MOESM17_ESM.jpg]

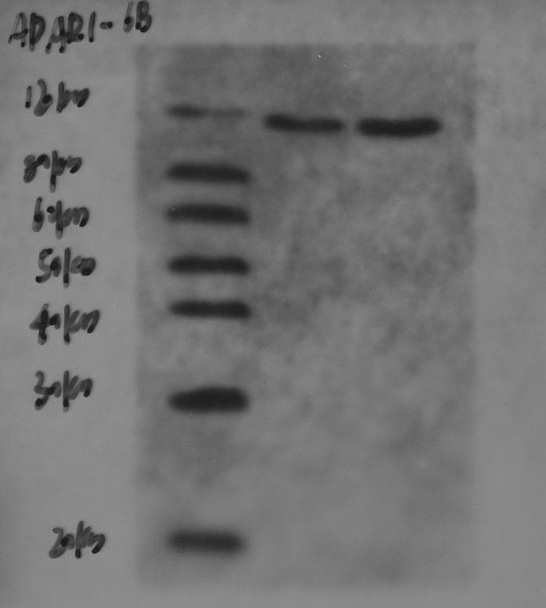

Supplement: Supplementary file 18 — Additional file 18. Full-length gel images of fig6B-ADAR1. [file 12872_2021_2438_MOESM18_ESM.jpg]

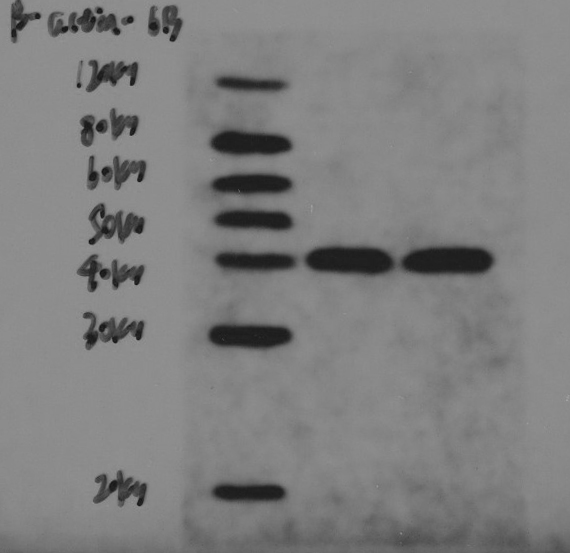

Supplement: Supplementary file 19 — Additional file 19. Full-length gel images of fig6B-β-actin. [file 12872_2021_2438_MOESM19_ESM.jpg]

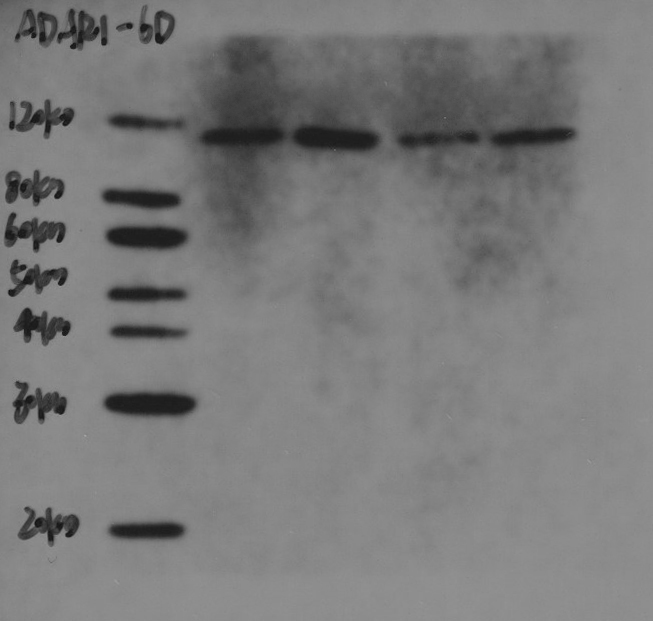

Supplement: Supplementary file 20 — Additional file 20. Full-length gel images of fig6D-ADAR1. [file 12872_2021_2438_MOESM20_ESM.jpg]

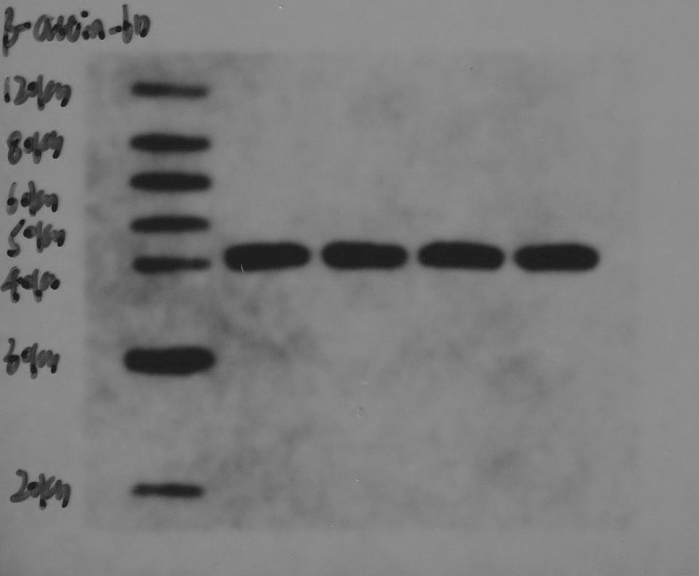

Supplement: Supplementary file 21 — Additional file 21. Full-length gel images of fig6D-β-actin. [file 12872_2021_2438_MOESM21_ESM.jpg]

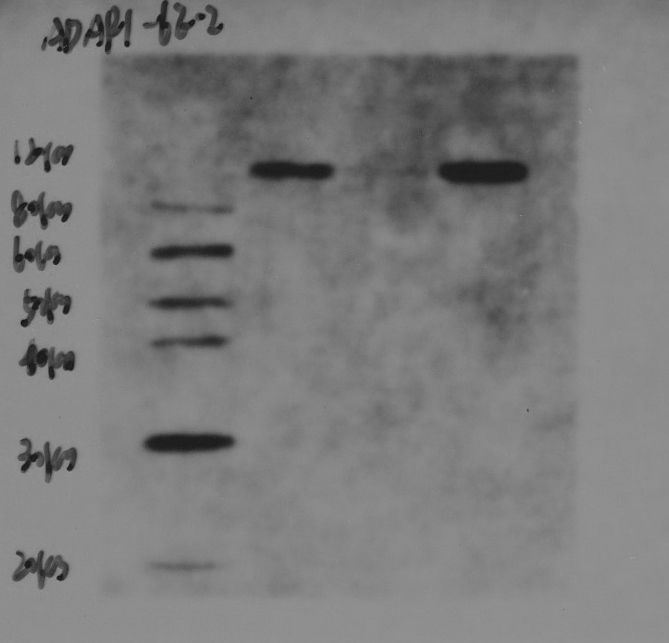

Supplement: Supplementary file 22 — Additional file 22. Full-length gel images of fig6E-ADAR1-H9c2. [file 12872_2021_2438_MOESM22_ESM.jpg]

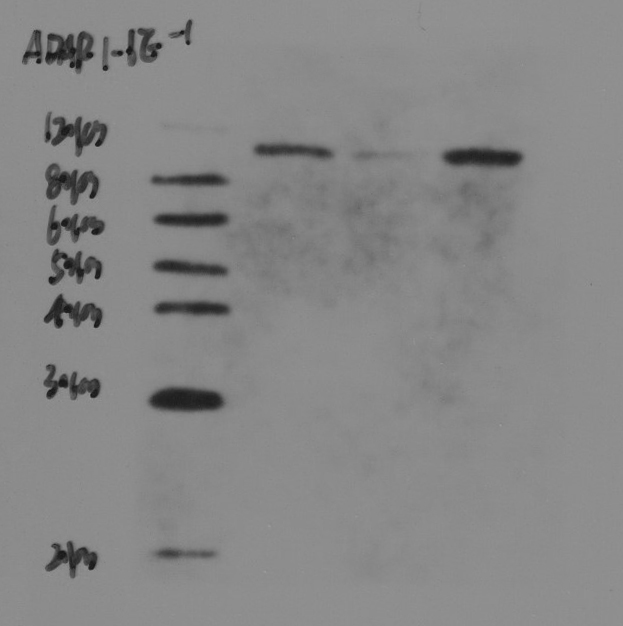

Supplement: Supplementary file 23 — Additional file 23. Full-length gel images of fig6E-ADAR1-primary. [file 12872_2021_2438_MOESM23_ESM.jpg]

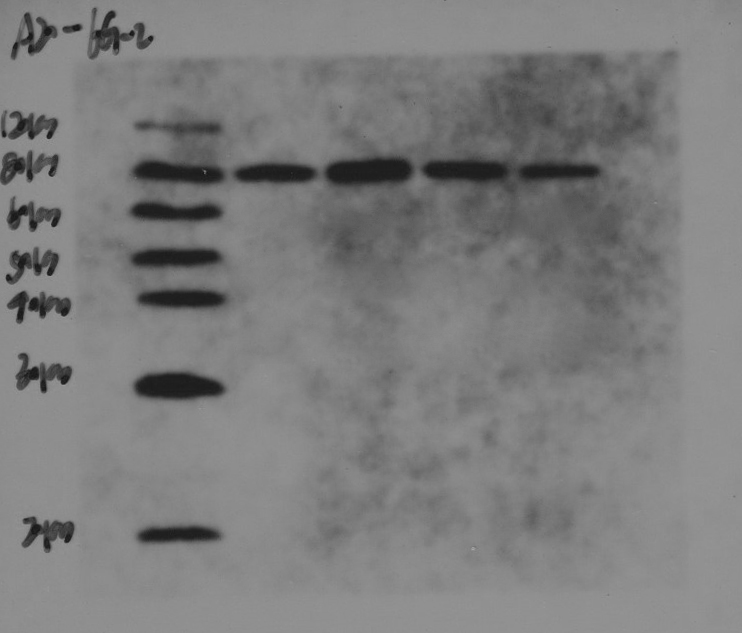

Supplement: Supplementary file 24 — Additional file 24. Full-length gel images of fig6G-A20-H9c2. [file 12872_2021_2438_MOESM24_ESM.jpg]

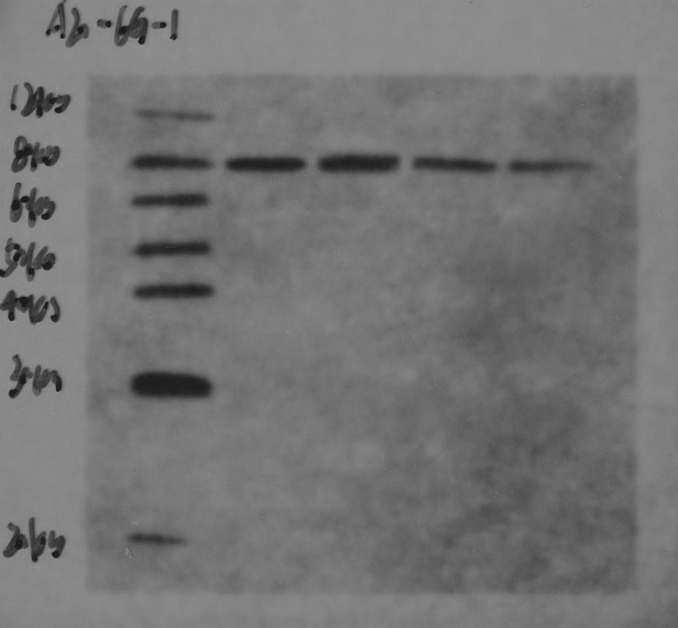

Supplement: Supplementary file 25 — Additional file 25. Full-length gel images of fig6G-A20-primary. [file 12872_2021_2438_MOESM25_ESM.jpg]

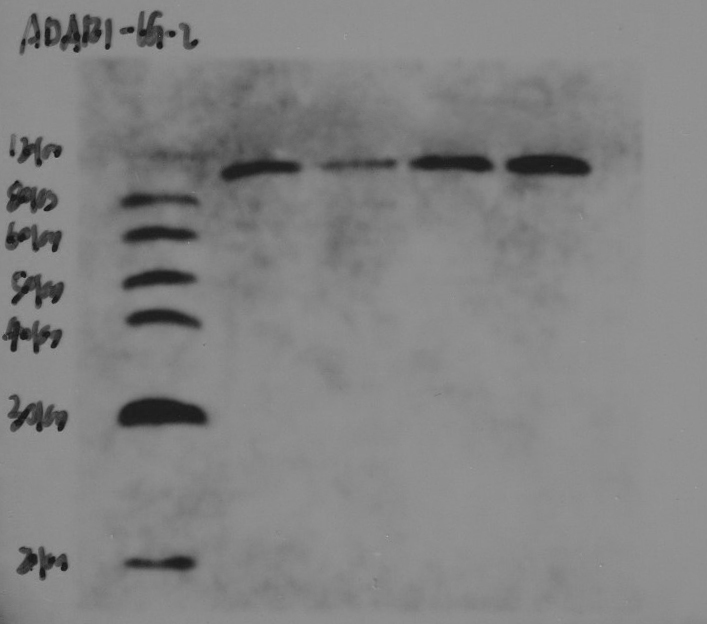

Supplement: Supplementary file 26 — Additional file 26. Full-length gel images of fig6G-ADAR1-H9c2. [file 12872_2021_2438_MOESM26_ESM.jpg]

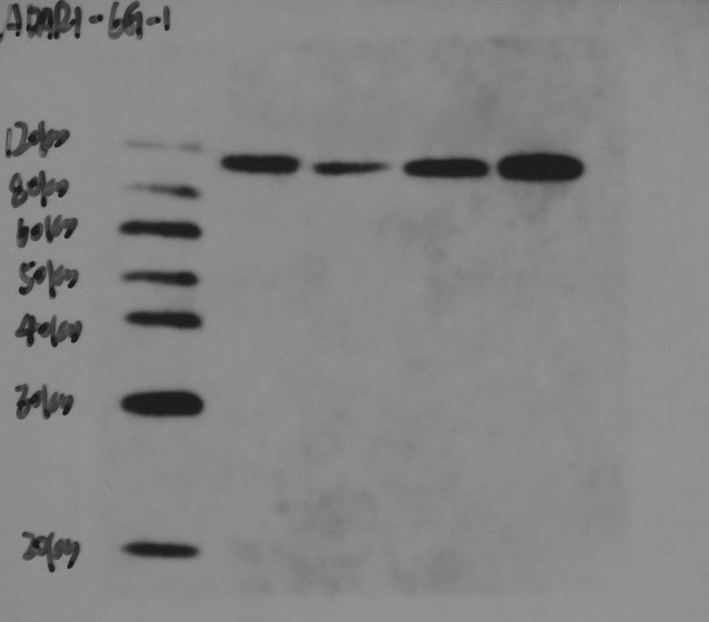

Supplement: Supplementary file 27 — Additional file 27. Full-length gel images of fig6G-ADAR1-primary. [file 12872_2021_2438_MOESM27_ESM.jpg]

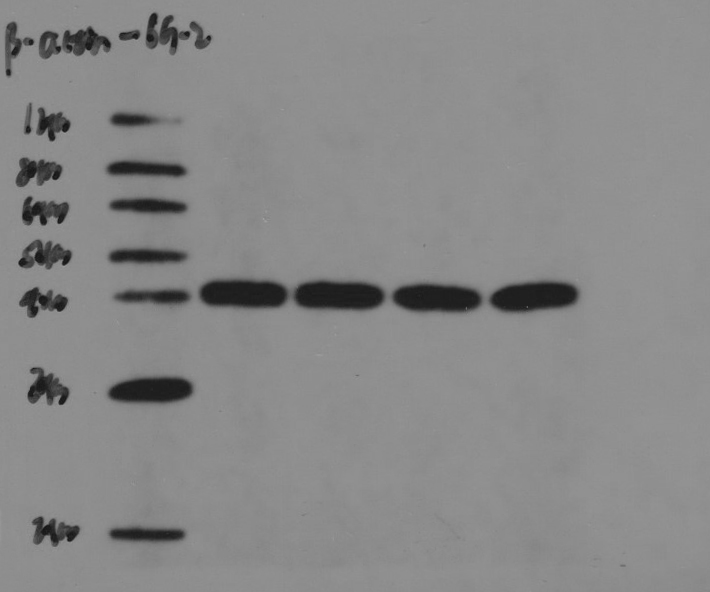

Supplement: Supplementary file 28 — Additional file 28. Full-length gel images of fig6G-β-actin-H9c2. [file 12872_2021_2438_MOESM28_ESM.jpg]

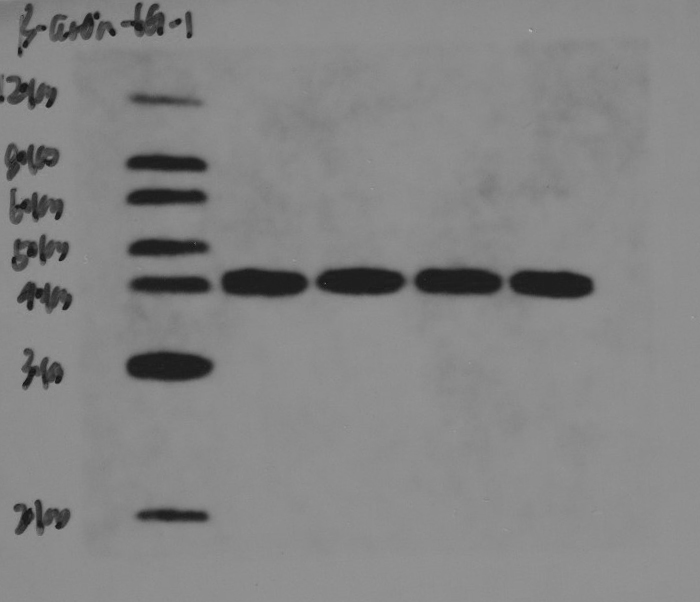

Supplement: Supplementary file 29 — Additional file 29. Full-length gel images of fig6G-β-actin-primary. [file 12872_2021_2438_MOESM29_ESM.jpg]
